# MTFN: multi-temporal feature fusing network with co-attention for DCE-MRI synthesis

**DOI:** 10.1186/s12880-024-01201-y

**Published:** 2024-02-19

**Authors:** Wei Li, Jiaye Liu, Shanshan Wang, Chaolu Feng

**Affiliations:** 1 Key Laboratory of Intelligent Computing in Medical Image MIIC, Northeastern University, Shenyang, China; 2 School of Computer Science and Engineering, Northeastern University, Shenyang, China

**Keywords:** DCE-MRI, Medical image synthesis, Co-attention, Multi-temporal feature fusing, Hybrid features

## Abstract

**Background:**

Dynamic Contrast Enhanced Magnetic Resonance Imaging (DCE-MRI) plays an important role in the diagnosis and treatment of breast cancer. However, obtaining complete eight temporal images of DCE-MRI requires a long scanning time, which causes patients’ discomfort in the scanning process. Therefore, to reduce the time, the multi temporal feature fusing neural network with Co-attention (MTFN) is proposed to generate the eighth temporal images of DCE-MRI, which enables the acquisition of DCE-MRI images without scanning. In order to reduce the time, multi-temporal feature fusion cooperative attention mechanism neural network (MTFN) is proposed to generate the eighth temporal images of DCE-MRI, which enables DCE-MRI image acquisition without scanning.

**Methods:**

In this paper, we propose multi temporal feature fusing neural network with Co-attention (MTFN) for DCE-MRI Synthesis, in which the Co-attention module can fully fuse the features of the first and third temporal image to obtain the hybrid features. The Co-attention explore long-range dependencies, not just relationships between pixels. Therefore, the hybrid features are more helpful to generate the eighth temporal images.

**Results:**

We conduct experiments on the private breast DCE-MRI dataset from hospitals and the multi modal Brain Tumor Segmentation Challenge2018 dataset (BraTs2018). Compared with existing methods, the experimental results of our method show the improvement and our method can generate more realistic images. In the meanwhile, we also use synthetic images to classify the molecular typing of breast cancer that the accuracy on the original eighth time-series images and the generated images are 89.53% and 92.46%, which have been improved by about 3%, and the classification results verify the practicability of the synthetic images.

**Conclusions:**

The results of subjective evaluation and objective image quality evaluation indicators show the effectiveness of our method, which can obtain comprehensive and useful information. The improvement of classification accuracy proves that the images generated by our method are practical.

## Introduction

In recent years, medical images, such as positron emission tomography (PET), computed tomography (CT) and magnetic resonance images (MRI), occupy an important position in clinical application, which provide a lot of information fordisease diagnosis [1–3]. MRI is widely used in the diagnosis of diseases because of its noninvasive as it can provide images with different parameters, such as dynamic contrast enhanced MRI (DCE-MRI), diffusion weighted imaging (DWI) and T1/T2 [4–6]. The MRI images with different parameters can provide different relevant information of the disease.

Due to the high resolution of DCE-MRI, it is often used as the main mode in the diagnosis and treatment of clinical diseases. DCE-MRI has a total of eight time series, the first of which is the images without contrast agent and the other time series are acquired at intervals after the addition of contrast agent. In medicine, the first, third and eighth temporal images of DCE-MRI are of great significance. These three temporal images have potential anatomical information, combining multi temporal images can provide more comprehensive information. The collection of eight temporal images of DCE-MRI requires a long scanning time [7–10]. The patients don’t feel well during this complete scan. It is very meaningful to shorten the scanning time as much as possible and simplify the breast MR scanning sequence.

Medical image synthesis aims to automatically extract features from existing medical images and synthesize target images. It is essential for modern computer-aided diagnosis (CAD) applications, such as disease screening and diagnosis. It can synthesize another modality images from one modality and not only solve the problem that deep learning requires a large amount of data and medical image data is less or missing, but also solve the possible adverse effects in the imaging process [11]. For example, due to the problems of poor image quality and data loss, Cheng, et al. designed an excellent network structure (HiNet), which realizes the synthesis of multi-modal images [12]. Wolterink JM et al. synthesized CT images by using MRI images through the CycleGAN model [13], which solves the problems that CT scanning is expensive, increases the burden on patients and is easy to cause harm to human body in the process of CT scanning. Standard dose PET is synthesized through low dose PET, so as to reduce artifacts and noise in low dose pet and avoid harm to human body caused by standard dose PET [14].

However, most of the existing synthesis research methods are single temporal images synthesis target temporal images or use multi temporal images feature fusion to generate images, the relationship between corresponding pixels can be obtained by simple fusion, the information interaction between different modal images is ignored and the relationship between modes is not fully utilized. And in the existing multi-modal synthesis methods, the unique features of single modality are often ignored.

How to fully integrate the features of multi temporal images to generate more realistic real images is a challenge. Using multi temporal images to obtain comprehensive information improves the accuracy of disease diagnosis. Therefore, to explore their rich pixel information and find the potential correlation between them to improve various medical tasks, we use the Co-attention module to obtain the information interaction of multi-scale features of multi temporal images and obtain the remote dependence between pixels, which is conducive to GAN to generate higher quality images. The contributions of this paper are as follows:

1. We propose a novel network (MTFN) to realize the multi temporal image fusion of DCE-MRI and generate the eighth temporal image. Experiments prove the effectiveness of our method, and verify that in the synthesis, DCE-MRI multi temporal image feature fusion generates eighth temporal images better than single temporal image generation.
2. The Co-attention module is applied to the feature fusion of multi temporal images. We use Co-attention module to obtain the shared features of the two temporal images, and fully integrate the unique and shared features of each temporal images to obtain comprehensive information.
3. We integrate the synthesis task of DCE-MRI multi temporal images and the classification task of DCE-MRI molecular typing of breast cancer, used two types of evaluation indicators to comprehensively evaluate the performance of our method.

The main structure of this paper is as follows: the second part mainly introduces the related work, the third part introduces our method and network components in detail, the fourth part is the experimental setup, including datasets, evaluation indicators and other related contents and the fifth part is the experimental results. Finally is the conclusion.

## Related work

In 2014, GAN was first proposed by Goodflow et al. [15], an excellent generative model trained by the method of confrontation learning. GAN is composed of generator network and discriminator network. The generator network can generate sample data approximately distributed with the input sample, and the discriminator network can judge the authenticity of the sample, which will correctly judge the real samples and the samples generated by the generator as much as possible. The discriminator network is essentially a binary classification network. Both of them can improve the performance of the generator and discriminator through confrontation learning. GAN is used in various fields of medicine such as classification, denoising, reconstruction and synthesis [16–18] and has achieved great success [19, 20]. In recent years, researchers have proposed many improved GAN models [21].

LAN et al. proposed that 3D GAN synthesize MRI images through PET images [22], Bmgan can synthesize PET from brain, which can preserve brain structure well [23]. Wang et al. proposed Ea-GANs based on edge perception, which adds additional constraints to the similarity between the real edge map and synthesized edge map. Ea-GANs pays more attention to the edge information of the image and generates high-quality images [24]. Wu et al. develop an encoder-decoder neural network with residual inception block to synthesis breast images [25]. M. Yurt et al. synthesized images with Multi-Contrast MRI through cycleGAN and pix2pix model to obtain more diagnostic information [26]. Multi-modality glioma MRI synthesis method is designed, which can obtain the common feature space and lesion-specific representations [27]. Zhou et al. proposed HiNet to synthesize the target modality images and Mixed Fusion Block is used to fuse features, which contains element-wise summation, element-wise product and element-wise maximization to fuse the features of each mode [28]. Luo et al. proposed EP_IMF-GAN with iterative multi-scale feature fusion and the MRI synthesis method can preserve edge information by generating the edge image of the target modality as an auxiliary condition [29]. Shen et al. use the channel-wise attention and spatial attention to get the target feature map, which makes the effective learning of the image synthetic model [30].

## Method

**Fig. 1:**
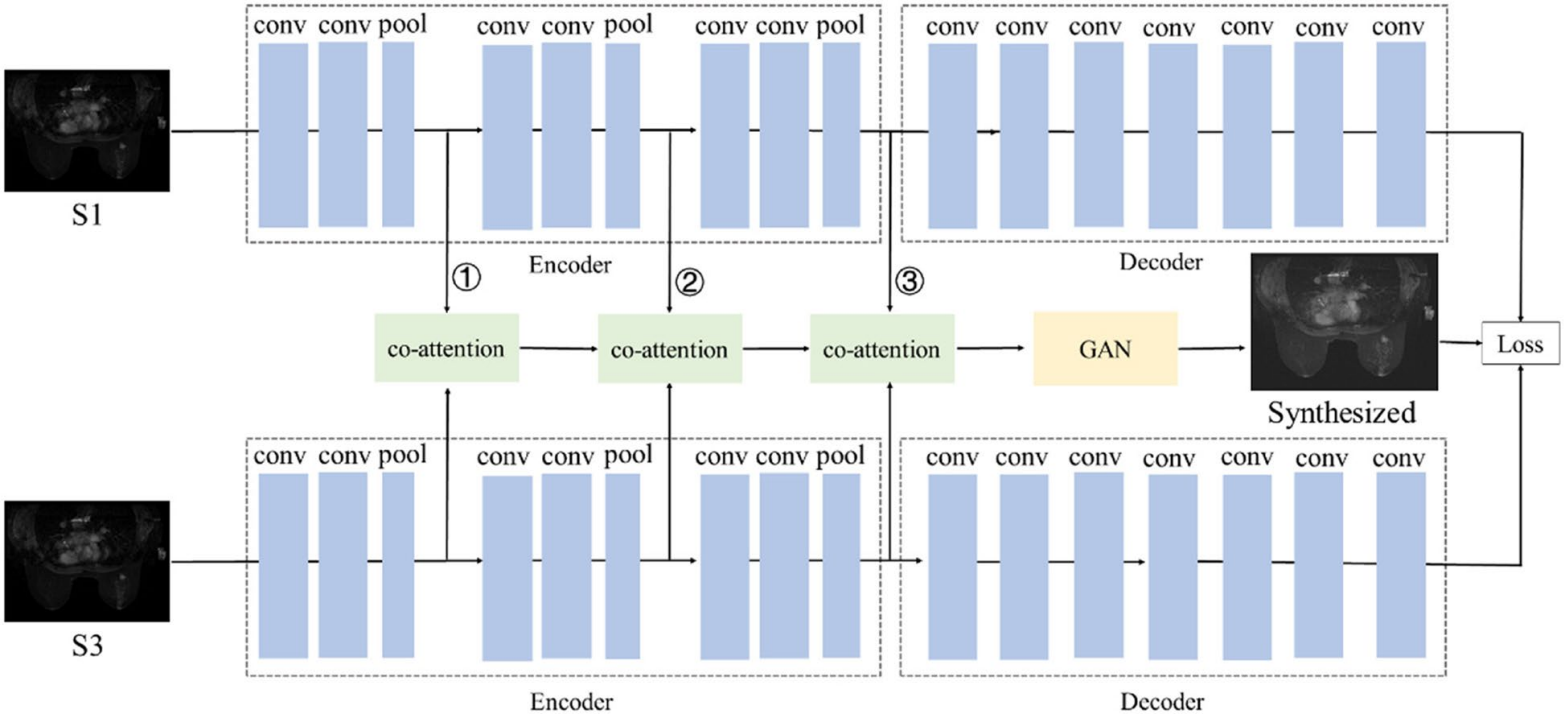
The network structure of MTFN

The network structure (MTFN) can be divided into three parts: feature extraction network of single temporal image, Co-attention module and eighth temporal image synthesis network. The single temporal image feature extraction network is mainly used to obtain the unique features of the first phase and the third phase. To obtain more comprehensive mixed features, Co-attention module is used to fully fuse the features of the first and the third temporal images, which are extracted in different layers of the feature extraction network. The features include both the unique features of single temporal images and the shared features of multi temporal images, which are used as the input of the synthesis network. Finally the eighth temporal images are synthesized. The flow chart of this architecture is shown in Fig. 1.

### Feature extraction network of single temporal image

The first and the third temporal image feature extraction network have the same network structure. The feature extraction network of single temporal image is composed of three encoder blocks and decoder, which is similar to the Unet structure without skip connection. An encoder block consists two 3 × 3 convolutional layers and one maxpool operation. The decoder consists of 3 × 3 convolution layer. The activation function used in the last layer of the network is Tanh, others are ReLU. After each encoder block in the network, we will get the feature maps of the first and third temporal images. The feature maps of the two temporal images will be fused through Co-attention module, and the feature maps will also be added to the generator.

In the process of image generation, in addition to the common features of multiple temporal images, the unique features of each temporal images are also very important. Therefore, we have reconstructed the images, and the reconstruction loss value is a part of the total loss. The objective function of reconstruction is as follows, where ${S}_{i}$ represents the original image and ${\widehat{S}}_{i}$ represents the reconstructed image.

1 $${\mathcal{L}}_{{R}_{i} }=\sum {\Vert {S}_{i}-{\widehat{S}}_{i}\Vert }_{1}$$

### Co-attention module

**Fig. 2:**
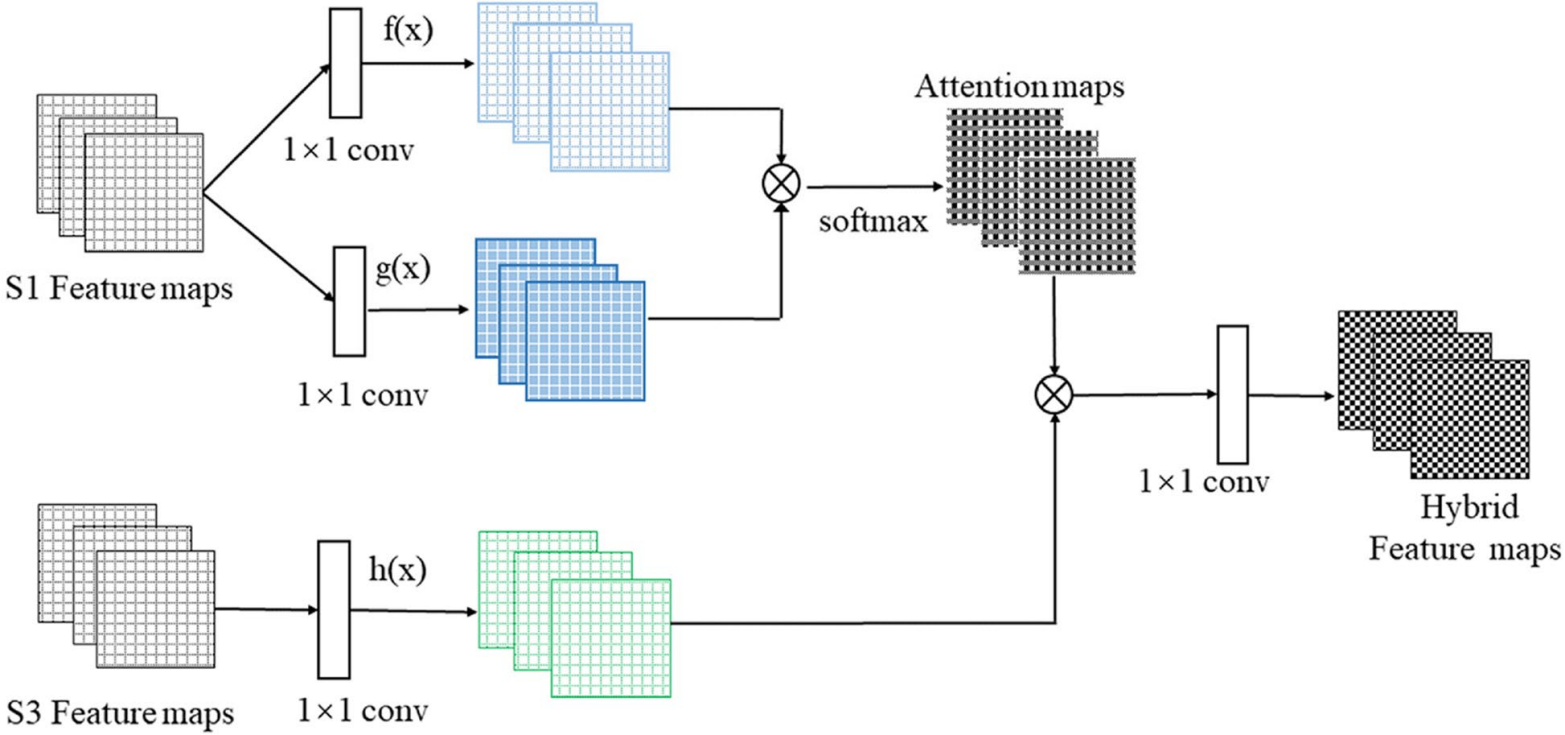
The architecture of the Co-attention module

In the existing fusion operations, it is often only to fuse the features at the pixel level, such as simple addition and averaging of the corresponding pixels, which can not obtain the remote dependency between the pixels. Therefore, in order to explore the position relationship of pixels between two different temporal images, we introduce Co-attention module into the feature fusion of two temporal images, capture the dependency between the features of two temporal images and the hybrid features are obtained. The architecture of the model is shown in Fig. 2.

First, the feature maps of the first temporal images are passed through 1 × 1 convolution operation, and then multiply the obtained feature maps, and softmax operation to obtain attention maps, so we obtain the positional relationship between each position of the first temporal feature map and the whole feature map, and capture the remote dependency. Second, to obtain feature dependency of the first and third temporal images, multiply attention maps and the feature maps which are the feature maps of the third temporal images through 1 × 1 convolution. Finally, after softmax, the final hybrid feature maps are obtained.

The features of single temporal images are captured through convolution layer, and the feature information between different scales of each phase is mined, and then feature fusion is carried out. According to the different location of fusion features, the usual feature fusion methods are divided into early fusion, middle fusion and late fusion. Early fusion is a basic fusion method, which directly fuses the signals output by each sensor, middle fusion is the direct fusion of feature extraction, and late fusion is the process of re fusion of feature recognition and classification results. In this paper, we experiment on different feature fusion methods to obtain the optimal fusion position.

### Eighth temporal image generation network

**Fig. 3:**
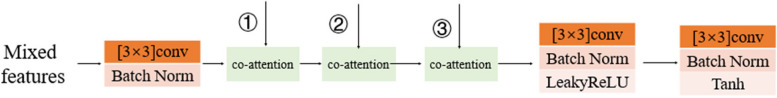
The architecture of the generator

**Fig. 4:**
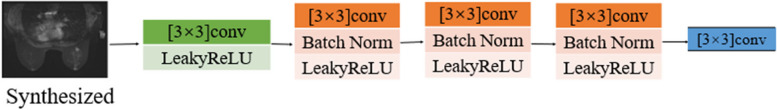
The architecture of the discriminator

We take the obtained hybrid features in the early stage as the input of the network, and use the important temporary image generation network to realize image generation. The network is a standard GAN network, which is composed of generator and discriminator. Its structure is shown in Figs. 3 and 4.

The generator generates as realistic images as possible, trying to deceive the discriminator. At the same time, the discriminator judges the generated image as false as possible, and we use PatchGAN structure as discriminator that its output is n × n matrix to evaluate the image generated by the generator. In our method, we set n = 6. In the process of network training, the generator and discriminator gamble each other, and finally generate realistic images. And loss function of the GAN and total objective function consists of three terms and can be written as

2 $$\begin{array}{l}{\mathcal{L}}_{\mathrm{GAN }}= {\mathbf{E}}_{{S}_{1},{S}_{3}\sim {p}_{{\text{data}} \, }}\left[{\text{log}}\left(1-D\left({\text{G}}\left({S}_{1},{S}_{3}\right)\right)\right)\right]\\ + {\mathbf{E}}_{{S}_{1},{S}_{3},{S}_{8}}\left[{\Vert {S}_{8}-{\text{G}}\left({S}_{1},{S}_{3}\right)\Vert }_{1}\right]\\ + {\mathbf{E}}_{{S}_{8}\sim {p}_{{\text{data}} \, }}\left[log(D({S}_{8}))\right]\end{array}$$

3 $${\mathcal{L}}_{{\text{Total}}}={\lambda }_{{R}_{1}}{\mathcal{L}}_{{R}_{1} }+{\lambda }_{{R}_{3}}{\mathcal{L}}_{{R}_{3} }+{{\lambda }_{{\text{GAN}}}\mathcal{L}}_{\mathrm{GAN }}$$

## Experimental setup

### Dataset

We use two datasets: the multi modal Brain Tumor Segmentation Challenge 2018 dataset (BraTs2018) and clinical breast DCE-MRI dataset from hospitals.

1. BraTs2018: The BraTs2018 dataset is used for multi modal brain tumor segmentation in MICCAI competition. It contains 285 patients. Each patient contains four modal datasets, namely T1, T1c, T2 and Flair. The image size is 240 × 240 × 155. In the experiment, we use the T1 and T2 modes to generate Flair.
2. Clinical Breast DCE-MRI dataset: The clinical breast DCE-MRI dataset contains 232 patients.

Each patient contains a data set of eight time series. The image size of each time series is 512 × 512 × 48. We generate the eighth temporal images through the first and the third temporal images.

### Evaluation metrics

Evaluate the quality of generated images to verify the effectiveness of the synthesis method that introduces the Co-attention module, we use the three commonly used evaluation indicators to measure the quality of synthetic images, and compare the performance of the method. The three evaluation indexes: PSNR (Peak signal-to-noise ratio), NMSE (Normalized Mean Squared Error) and SSIM (Structural Similarity Index Measurement).

PSNR represents the ratio of the maximum possible power of the signal to the destructive line noise power that affects its representation accuracy. The calculation formula is as follows, where m, n represents the size of the image, T (i, j) represents the pixels in the real image, and G (i, j) represents the pixels in the generated image.

4 $$MSE=\frac{1}{mn}{\sum }_{i=1}^{m}{\sum }_{j=1}^{n}||T(i,j)-G(i,j)|{|}^{2}$$

5 $$PSNR=10\ast\text{log}\;10\left(\frac{{MAX}^2\left[T\left(i,j\right),G\left(i,j\right)\right]}{MSE}\right)$$

NMSE represents normalized mean square error. The smaller the value, the better. The calculation formula is as follows:

6 $$NMSE=\frac{{\sum }_{i=0}^{m-1}{\sum }_{j=0}^{n-1}[T(i,j)-G(i,j){]}^{2}}{{\sum }_{i=0}^{m-1}{\sum }_{j=0}^{n-1}[T(i,j){]}^{2}}$$

The similarity of two images can be measured by SSIM. The calculation formula is as follows, and ${\mu }_{G}$ is the average value of the generated image, ${\mu }_{T}$ is the average value of the real image, ${\mu }_{G}^{2}$ is the variance of the generated image, ${\mu }_{T}^{2}$ is the variance of the real image, ${\sigma }_{GT}$ is the standard deviation between the generated image and the real image, ${c}_{1}$ and ${c}_{2}$ are constants respectively to avoid errors caused by denominator 0.

7 $$SSIM=\frac{(2{\mu }_{G}{\mu }_{T}+{c}_{1})({\sigma }_{GT}+{c}_{2})}{({\mu }_{G}^{2}+{\mu }_{T}^{2}+{c}_{1})({\sigma }_{G}^{2}+{\sigma }_{T}^{2}+{c}_{2})}$$

The above three evaluation indexes are used to evaluate the quality of the generated images. In addition, to verify the usefulness of the images generated by our method, we also used real images and generated images to classify breast cancer molecular subtypes which is helpful for doctors to formulate individualized treatment plans for patients. The evaluation indexes used for classification are Accuracy, Precision, Recall and F1 score.

### Implementation details

Each image in the BraTs2018 dataset and the private DCE-MRI dataset is divided into 4 × 128 × 128 and 25 × 128 × 128 patch images. All images are normalized and preprocessed. The preprocessed images are input into the network in pairs. The network parameters are updated through the total loss function value of a single phase network and the network synthesizing the final target image.

We use the pytorch framework for network construction, select Adam optimizer, the learning rate is set to 0.001, the trained batch size = 4. All experiments are carried out in NVIDIA GeForce GTX1080 Ti GPU.

## Result

In this paper, we have carried out three different tasks: explore the location of Co-attention fusion module in the network, so as to obtain the best fusion method. Through the advanced fusion method obtained from the previous implementation, we synthesize the eighth temporal images of DCE-MRI and compare it with the existing methods to verify the effectiveness of our method. The classification experiment of DCE-MRI breast molecular typing is carried out by using the generated image to further verify the practicability of the generated image.

### Different fusion positions

First we explore the way of feature fusion using private dataset and public dataset, and conduct early fusion, medium-term fusion and late fusion experiments respectively. The early fusion is the fusion operation before the first layer of the network and only one co-attention fusion. The middle fusion is the fusion operation in the process of extracting image features, and the late fusion is the fusion after the last layer of the network.

**Fig. 5:**
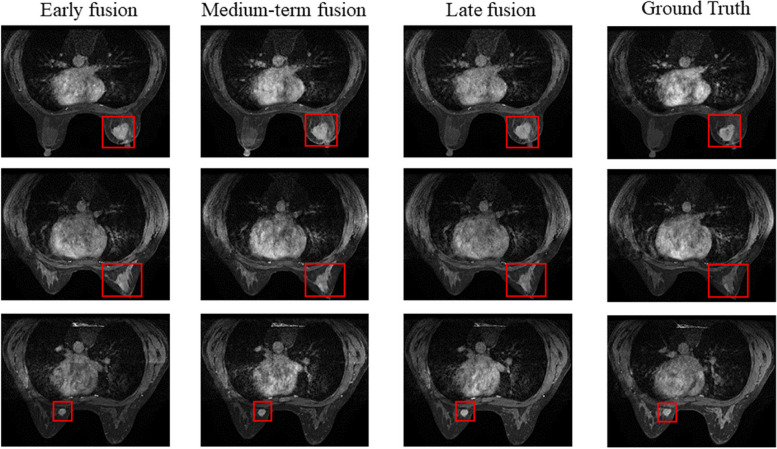
The generated eighth images of DCE-MRI using different way of feature fusion

**Fig. 6:**
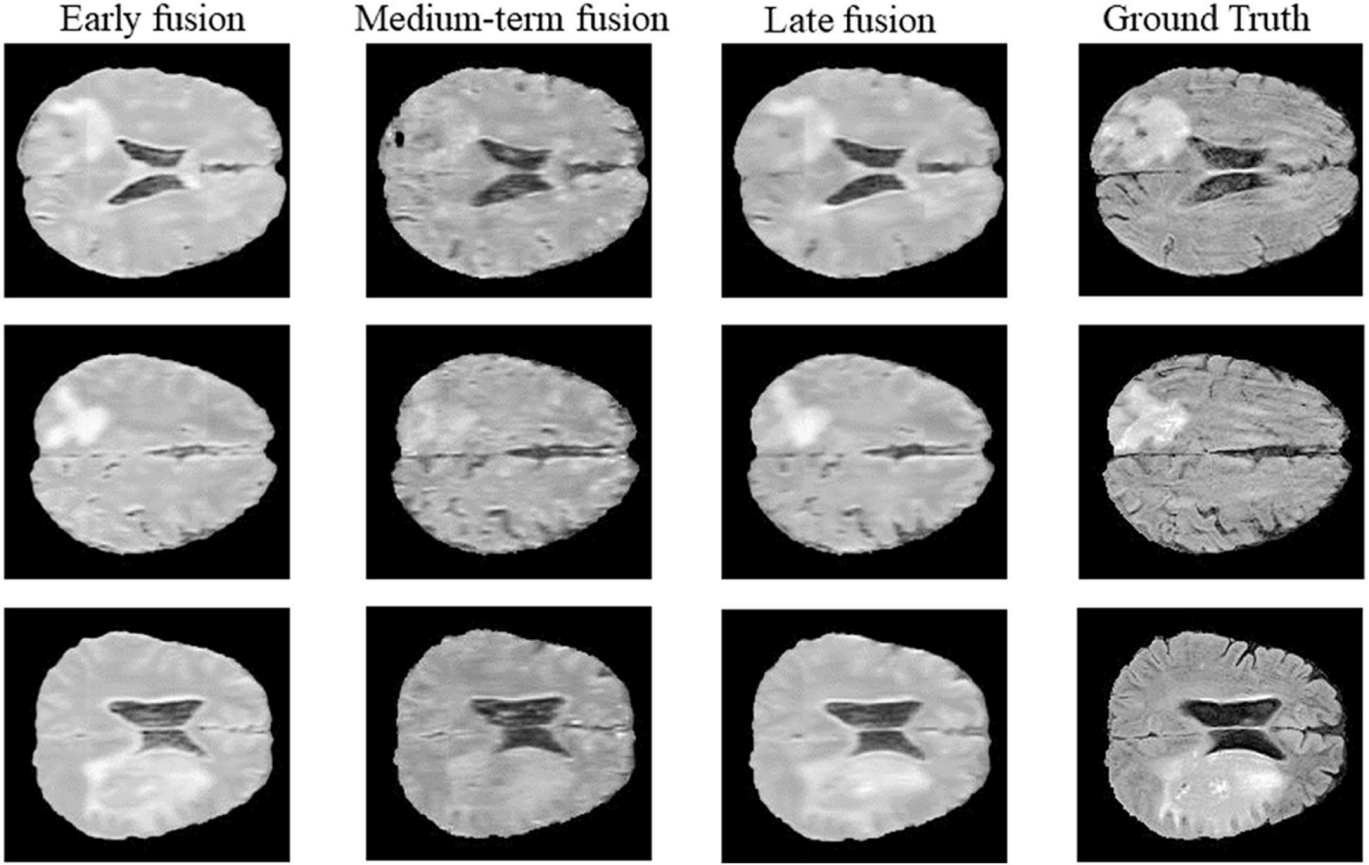
The generated Flair images of BraTs2018 using different way of feature fusion

The experimental results are shown in Table 1 and Table 2, the generated images are shown in Figs. 5 and 6. Experiments using our DCE-MRI dataset show that the generated images are similar to the real images, and the results of medium-term fusion are the best from the analysis of objective indicators. On BraTs2018 dataset, the images generated by early fusion and late fusion are fuzzy and smooth, and the images generated by medium-term fusion contain more texture details. Medium-term fusion is the best fusion way. Therefore, our method adopts the feature fusion method based on Co-attention and medium-term fusion.

**Table 1:** The results of the different way of feature fusion on DCE-MRI

Methods	DCE-MRI	PSNR↑	NMSE↓	SSIM↑
Early fusion	S1, S3 → S8	21.45	1.38	0.67
Late fusion	S1, S3 → S8	21.46	1.20	0.67
Medium-term fusion	S1, S3 → S8	**21.67**	**1.04**	**0.68**

**Table 2:** The results of the different way of feature fusion on BraTs2018

Methods	BraTs2018		PSNR↑	NMSE↓	SSIM↑
Early Fusion		T1, T2 → Flair	21.29	0.09	0.82
Late Fusion		T1, T2 → Flair	22.27	0.07	**0.83**
Medium-term fusion		T1, T2 → Flair	**22.41**	**0.07**	0.82

### Eighth temporal image synthesis

**Fig. 7:**
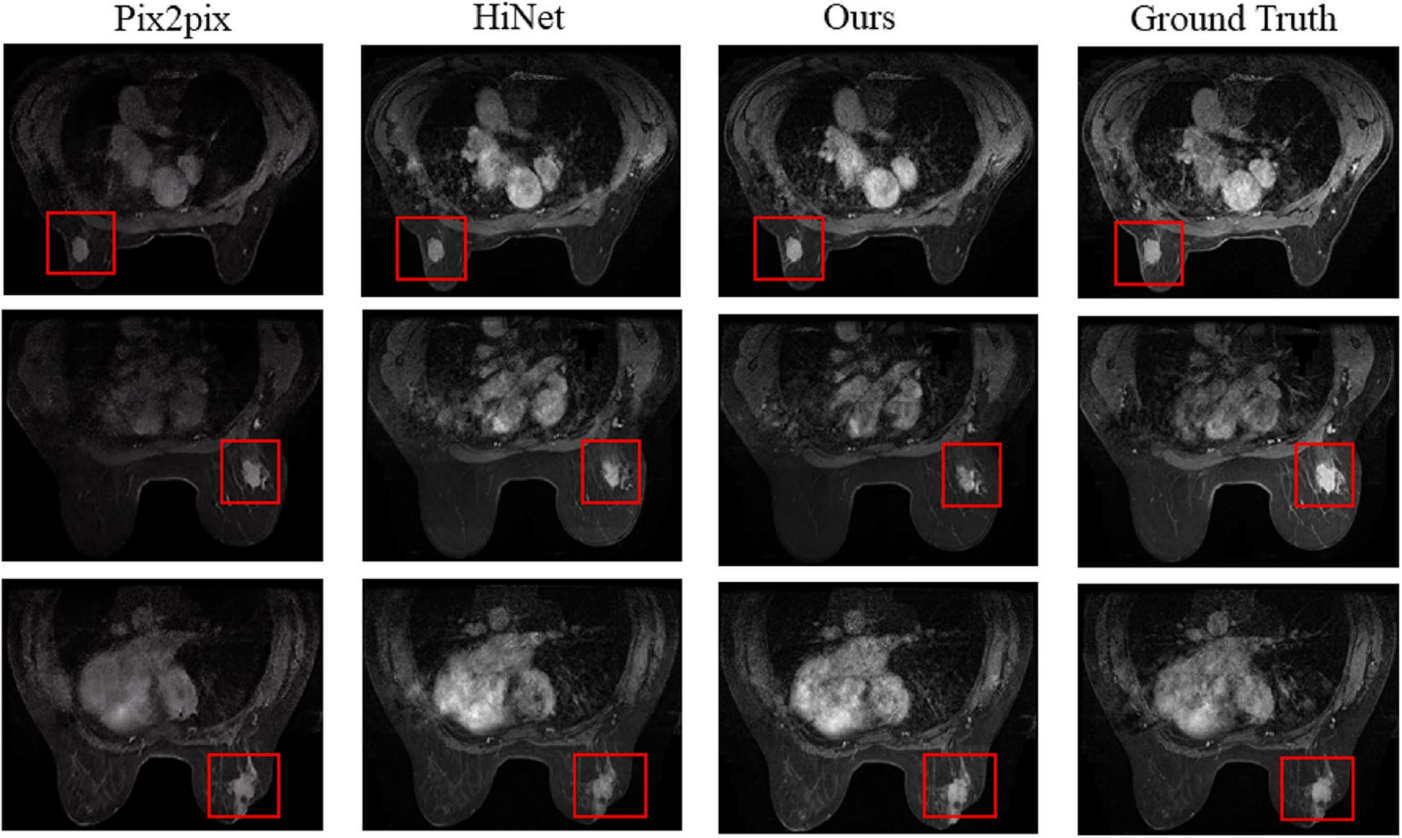
The generated eighth temporal images of DCE-MRI with three methods

**Fig. 8:**
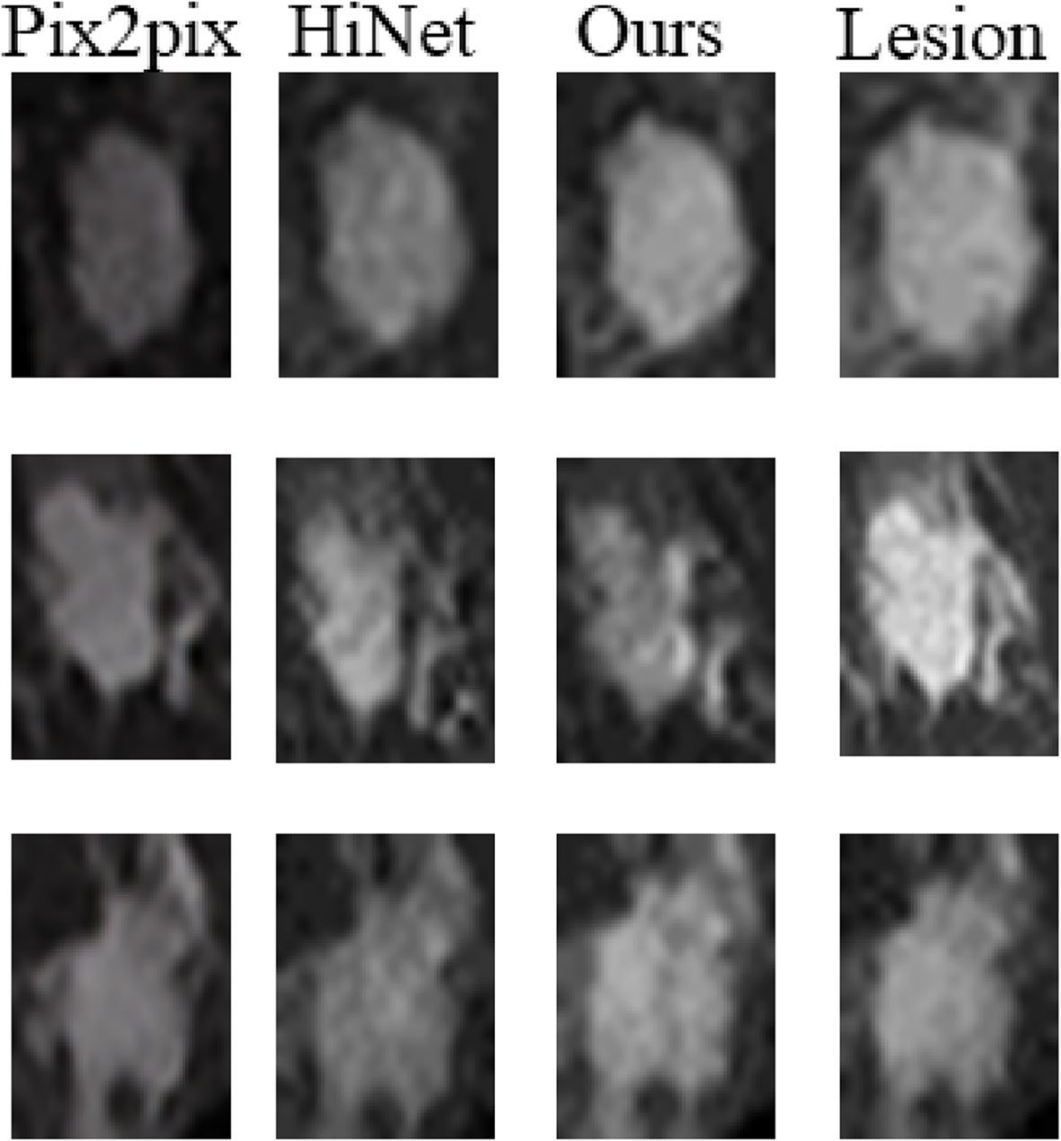
The generated lesion with three methods, compared to other methods, the generated lesion with our methods is more complete and realistic

We use Clinical dataset to do experiments with our method, and compare with the existing methods Pix2pix and HiNet. From the evaluation index of Table 3, the PSNR and SSIM obtained by our method are higher than those obtained by pix2pix and HiNet. NMSE is lower than that obtained by HiNet. On the whole, our method has also been improved. And the generated eighth temporal images are shown in Fig. 7, compared to real images, the eighth temporal images synthesized by pix2pix, HiNet and our method are basically consistent with the real images on the whole, but in terms of details, we truncate and enlarge the focus in the images, as shown in Fig. 8, the image generated by pix2pix and HiNet method is fuzzy and low contrast. The images generated by our method is more realistic and complete, such as the size and shape of the lesion.

**Table 3:** Compared with other methods on DCE-MRI breast images

Methods	DCE-MRI	PSNR↑	NMSE↓	SSIM↑
Pix2pix	S1, S3 → S8	18.53	**0.29**	0.66
HiNet	S1, S3 → S8	20.01	1.08	0.64
Ours	S1, S3 → S8	**21.67**	1.04	**0.68**

**Fig. 9:**
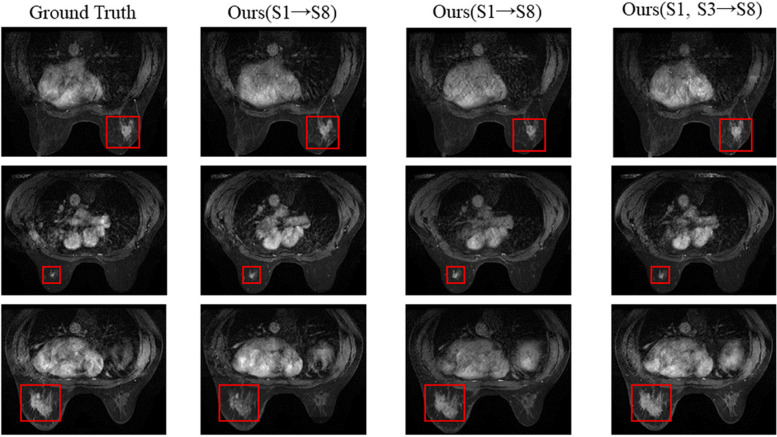
The generated eighth images with single temporal images and multi temporal images

Specifically, we use the single and multi temporal images of DCE-MRI to generate the eighth temporal images respectively, as shown in Table 4. The experimental results show that multi temporal images feature fusion can obtain more information and contribute to the generation of the eighth temporal images which are shown in Fig. 9.

**Table 4:** The result of fusion of single temporal images and multi temporal image to generate the eighth temporal images

Methods	DCE-MRI	PSNR↑	NMSE↓	SSIM↑
Ours	S1 → S8	21.58	1.06	0.67
Ours	S3 → S8	21.33	1.20	0.66
Ours	S1, S3 → S8	**21.67**	**1.04**	**0.68**

### Molecular typing of breast cancer in DCE-MRI

The experiment fully confirms the advanced nature of our method, and at the same time, in order to further verify the usefulness of the generated images, we used the original eighth temporal images and the generated eighth temporal images to conduct breast cancer molecular typing experiments which respectively, that is, non LuminalB and LuminalB molecular typing two classification. The experimental results are shown in Table 5. We first carried out classification experiments using the first and third temporal images, and then added the eighth temporal images for experiments. The experimental results show the necessity of the existence of the eighth temporal images, which is an indispensable series. Finally, the generated eighth temporal images are used to replace the original images for experiments.

**Table 5:** Results of molecular typing of DCE-MRI breast cancer on the First, Third, Eighth and Generated eighth temporal images by our method, which contains of two classes: non Luminal B and Luminal B

Data	Accuracy	Precision	Recall	F1-score
S1 + S3	83.55%	83.95%	83.70%	83.52%
S1 + S3 + S8	89.53%	90.44%	89.55%	89.46%
S1 + S3 + Generated_S8	**92.46%**	**93.06%**	**92.33%**	**92.40%**

In molecular typing experiments, as classifier, we adopt the DenseNet [20] as the base classifier to predict. The classification results are shown in Table 5 and the generated eighth temporal image improves the classification accuracy which fully prove that the image generated by our proposed method is practical.

## Conclusions

In this paper, we have proposed the MTFN based GAN and attention mechanism to realize the multi temporal image fusion of DCE-MRI and generate the eighth temporal image. The effectiveness of the image synthesis method of feature fusion with improved attention mechanism is verified on the BraTs2018 public dataset and DCE-MRI dataset. It is worth noting that we not only retain the multi-scale features of single temporal images, but also explore the correlation between two temporal images of DCE-MRI by introducing attention mechanism. The results show that this method can obtain synthetic images with clear structure, which not only simplifies the breast MR scanning sequence, but also has application value in clinical diagnosis.

At present, we realize the feature fusion of DCE-MRI multi-temporal images to generate the eighth-temporal images, and the accuracy of molecular classification of breast cancer using the generated eighth-temporal images is improved by 3%. Because the lesion information is more valuable for doctors’ diagnosis, we will consider local enhancement of lesion location in the next step to further improve the accuracy of classification.

## Data Availability

The datasets used and analyzed during the current study available from the corresponding author on reasonable request. We use two datasets: the multi modal Brain Tumor Segmentation Challenge 2018 dataset (BraTs2018) and clinical breast DCE-MRI dataset from hospitals. The BraTs2018 is introduced by Bjoern H. Menze et al. in The Multimodal Brain Tumor Image Segmentation Benchmark (BRATS) . The clinical data set involves patient privacy and is not convenient for disclosure.
